# Winter-ground microhabitat use by differently coloured phenotypes affects return rate in a long-distance migratory bird

**DOI:** 10.1007/s00442-024-05561-8

**Published:** 2024-05-09

**Authors:** Tiia Kärkkäinen, Keith A. Hobson, Kevin J. Kardynal, Toni Laaksonen

**Affiliations:** 1 Department of Biology, University of Turku, Turku, Finland; 2 Department of Evolutionary Ecology, National Museum of Natural Sciences, Madrid, Spain; 3 University of Western Ontario, London, Canada; 4 Environment and Climate Change Canada, Saskatoon, Canada

**Keywords:** Carbon-13, Colouration, Deuterium, Migratory connectivity, Nitrogen-15, Plumage, Return probability, Stable isotopes

## Abstract

Migratory bird populations are declining globally at alarming rates. Non-breeding site conditions affect breeding populations, but generalising non-breeding habitat conditions over large spatial regions cannot address potential fine-scale differences across landscapes or local populations. Plumage characteristics can mediate the effects of environmental conditions on individual fitness. However, whether different phenotypes use distinctive non-breeding sites, and whether they respond to non-breeding site conditions differently remains largely unknown. Stable isotopes (*δ*^13^C, *δ*^15^N, *δ*^2^H) of inert tissues are useful to infer habitat characteristics and geographic origins where those tissues were grown. We collected winter-grown feathers from pied flycatchers (*Ficedula hypoleuca*) on their breeding grounds over several years from males whose dorsal plumage colouration ranged continuously from brown to black and assessed their stable isotope values as proxies of local habitat conditions. Based on feather *δ*^2^H profiles we found that browner males spent their non-breeding season in drier habitats than black males. Assignment to origin analysis shows potential regional non-breeding ground separation between differently coloured males. High within-individual repeatability of both *δ*^13^C and *δ*^15^N indicate the pied flycatcher males return yearly to similar areas. Blacker males were more likely to return to the breeding grounds after dry years compared with brown males. The opposite was found in wet years. Our study demonstrates that different phenotypes are exposed to different non-breeding site conditions which can differentially affect individual survivorship. This has important ramifications for population dynamics under predicted climate change scenarios where especially brown phenotype pied flycatcher males may be under a risk of decreasing.

**Supplementary Information:**

The online version contains supplementary material available at 10.1007/s00442-024-05561-8.

## Introduction

Almost half of the world’s bird populations are declining (BirdLife International 2022) and migratory birds are declining faster than other groups (e.g. Laaksonen and Lehikoinen 2013; Runge et al. 2015). Migratory birds move vast distances annually between their breeding and non-breeding grounds to make use of the most suitable habitats and resources year-round (Alerstam et al. 2003). However, this makes them especially susceptible to changes in environmental conditions throughout their annual cycle (Ponti et al. 2020). Long-distance migrants especially spend only a short part of the annual cycle on their breeding grounds, thus uncovering factors that affect their survival during the non-breeding season provides crucial information needed to focus conservation efforts (Marra et al. 2015). Climatic conditions experienced during one part of the annual cycle can affect multiple demographic measures including individual survival (Newson et al. 2009; Pearce-Higgins et al. 2015). For example, precipitation levels and vegetation productivity on the non-breeding grounds have been associated with breeding population sizes of many migrant bird species (e.g. Ockendon et al. 2014; Schaub et al. 2005), although interpretation of the evidence has not always been straightforward (e.g. Beresford et al. 2019; Ockendon et al. 2014; Salewski et al. 2013). Indeed, estimating constant non-breeding site environmental conditions at large spatial scales may mask potential fine-scale differences in conditions experienced within regional non-breeding populations (Ishong et al. 2022).

Within populations, phenotypic differences may ultimately determine how environmental variables affect demographically important processes such as survival or sexual selection. In birds, breeding plumage coloration and quality can vary and lead to differential fitness among individuals (Dunn et al. 2015). Many bird species exhibit notable variation in colouration that can be explained by differences in genetic factors and in individual plasticity to environmental conditions (Roulin and Ducrest 2013). The existence of such variation indicates temporal and/or spatial changes in the direction of selection such as fluctuating selection for a specific phenotype (Bell 2010). This differential selection can lead to alternative phenotypes being associated with different habitats or to different abilities among phenotypes to cope with changing environmental conditions (Kassen 2002). For example, Gloger’s rule predicts that darker integuments are favoured in wetter (more humid) environments and lighter integument when conditions are drier (Gloger 1833). Evidence suggests that non-breeding habitat can have marked effects on individual fitness through carry-over effects (Marra et al. 1998; Norris et al. 2004; Reneerkens et al. 2020). Unfortunately, little is known about how different phenotypes are distributed within a species’ non-breeding range due to inherent difficulties in evaluating spatial migratory connectivity at fine spatial scales (Webster et al. 2002).

Measurements of naturally occurring stable isotopes in tissues have proven valuable in assessing migratory connectivity due to their ready application to each captured individual compared to the use of extrinsic markers such as GPS trackers that still pose substantial size and cost constraints (Hobson 1999a; Costa-Pereira et al. 2022), and which rarely can be used to infer local conditions mediated by diet. Tissue stable isotope measurements also provide a proxy for habitat characteristics during the period of tissue formation (Hobson 1999a). In particular, the stable isotopes of carbon (*δ*^13^C), nitrogen (*δ*^15^N), and hydrogen (*δ*^2^H) measured in feathers of birds provide useful information on moult origins and conditions as these isotopes are associated with vegetation types and land use as well as predictable spatial isotopic distributions or ‘isoscapes’ and all of them are expected to increase in drier conditions (Kelly 2000; Hobson et al. 2012b; Hoenig et al. 2021). In terrestrial systems, values of *δ*^13^C differentiate between primary producers with C_3_, C_4_ or CAM photosynthetic pathways (Bender 1968). Nitrogen stable isotope ratios (*δ*^15^N) are useful indicators of trophic position as they typically increase in a stepwise fashion with increasing trophic level (Hobson and Welch 1992; Hobson et al. 1994). Stable nitrogen isotope values can also be influenced by land-use practices and the use of fertilizers (Hobson 1999b; Hoenig et al. 2021). Tissue *δ*^2^H values are strongly associated with precipitation *δ*^2^H values, indicating connection between *δ*^2^H values and local rainfall (Hobson et al. 2012b). Thus, such isotopic markers in feathers can be used to infer provenance and act as an indicator of mesic vs. xeric conditions during feather growth (Marra et al. 1998; Hobson et al. 2012b; Vander Zanden et al. 2016; López-Calderón et al. 2017; van Wijk et al. 2021).

We investigated linkages between breeding plumage darkness and spatial origins and inferred environmental conditions on the non-breeding grounds in an Afro-Palaearctic migratory passerine, the pied flycatcher (*Ficedula hypoleuca*), by sampling feathers grown on their African non-breeding grounds. The pied flycatcher is a sexually dimorphic, insectivorous migratory passerine that undergoes a complete moult on the breeding grounds before autumn migration when males also change into a more cryptic, female-like plumage. A partial prenuptial moult occurs before spring migration in Africa when males moult into their conspicuous breeding plumage (Salewski et al. 2004; Jenni and Winkler 2020) with extensive variation in breeding plumage traits among males (Laaksonen et al. 2015). The colour of the dorsal plumage varies from almost completely black to almost completely brown closely resembling female *Ficedula* flycatchers breeding in sympatry (heterospecific mimicry; Calhim et al. 2014). Blackness in male plumage in breeding populations increases with increasing distance to Central Europe, while Central European males are mostly brown (Laaksonen et al. 2015). The dorsal colour is derived from melanin pigments which are strongly genetically regulated (Roulin and Ducrest 2013). The dorsal coloration of male pied flycatcher is heritable (Lehtonen et al. 2009) as well as highly repeatable within individuals (Järvistö et al. 2016). Males, regardless of colour, tend to become slightly darker after occupying dry non-breeding site conditions (Järvistö et al. 2016). While the conspicuous black plumage has been suggested to be sexually selected (Røskaft and Järvi 1983), evidence is mixed and instead it seems that fluctuating selection induced by changing temperatures during breeding maintain differently coloured pied flycatcher males in sympatry (Sirkiä et al. 2010; reviewed in Sirkiä and Qvarnström, 2021). Recently, Selonen et al. (2021) and Nater et al. (2022) showed using long-term datasets that large-scale population dynamics of pied flycatchers breeding in Finland and Great Britain are largely dependent on conditions experienced over the non-breeding period, therefore calling for the focus of studies and conservation efforts on migration routes and stationary non-breeding (hereafter, wintering) areas.

We used feathers collected annually as a part of a long-term study at a breeding site in Finland to infer the local conditions during moult in Africa in the full range of differently coloured individuals spanning eight years. We used stable isotope ratios (*δ*^13^C_f_, *δ*^15^N_f_, *δ*^2^H_f_) of winter-grown feathers as a proxy of local, isotopically different habitats in Africa. Our objectives were: (1) to investigate potential differences in wintering-site use among male pied flycatchers varying in plumage coloration (i.e. blackness), and (2) to model effects of local wintering site and more general African winter conditions on the return probability of differently coloured males to the breeding grounds. We predicted that (1) blacker individuals would show lower isotope values indicating mesic habitats (Gloger 1833; Salewski et al. 2002b; Roulin 2004; Hobson et al. 2012b), and (2) individuals exhibiting lower isotope values and/or blacker plumage would show higher return rates than individuals showing higher isotope values, especially after overall dry winters (Järvistö et al. 2016) due to differences in rainfall across sites (Hobson et al. 2012b).

## Materials and methods

### Data collection

Feather samples were collected from a pied flycatcher population breeding on the island of Ruissalo in Turku, Finland during the years 2007–2014. The long-term work and sampling were approved by the Animal Experiment Board in Finland (LOS-2007-L-264-254; ESAVI-2010-05480/Ym-23). The study site included 230 nest boxes before 2011, after which the area was expanded to 436 nest boxes (inner bottom area: 144 cm^2^, entrance hole Ø: 32 mm) while maintaining the same sites in the original area. In Finland, pied flycatchers arrive to the breeding grounds in early May (Velmala et al. 2015) and males were captured between 02 May and 20 July as part of a long-term monitoring study. All birds were fitted with a uniquely numbered aluminium ring and aged either as second calendar year (i.e. 1-year old) or older (≥ 2 years) based on feather characteristics (Svensson 1992). The middle tertial feather from one wing was collected from each bird and used later for stable isotope analyses. This feather is moulted during the pre-nuptial moult on the wintering grounds in Africa (Svensson 1992; Salewski et al. 2004). The same feather was collected and same region of the feather analysed for stable isotopes in all cases (Smith et al. 2008). In each year, ~ 30 males (28 males in 2013; 29 males in 2012; 30 males in 2007, 2008, 2014; 31 males in 2009, 2010, 2011; 240 samples in total) representing a range in plumage colouration from fully brown to fully black were picked for feather stable isotope analyses. We note that the selected individuals do not to represent the phenotype distribution of the population, which varies annually (Sirkiä et al. 2013), since for the purpose of this study the idea was to have a balanced sample of the coloration range in each year. Colouration of each individual was visually estimated at the field as the approximate proportion of black feathers in the dorsal plumage (areas in the head and back excluding the rump) and reported in percentages from 0 to 100% (Järvistö 2016) in 5% intervals (with 1% exceptions in both extremes). Colouration assessments were done by several people over the years. However, new people were always trained by an experienced investigator to assess colouration, and the repeatability of separate colour measurements have previously been found to be high (*r* = 0.88, *P* < 0.001 in (Järvistö et al. 2015)). Thus, we have no reason to believe that the colour measurements in this study would be strongly influenced by the assessor. We also assumed that any isotopic differences that may be linked to differential melanin content in feathers were relatively minor compared to differences anticipated among xeric vs mesic habitats in Africa. Evidence for this assumption was provided by Michalik et al. (2010) who found minor isotopic effects in feathers for *δ*^13^C and *δ*^15^N values, but, to our knowledge, similar investigations have not been performed for *δ*^2^H values. Nonetheless, the work of Hobson et al. (2012b) using feathers from numerous species in North America with varying feather coloration has shown a strong influence of local precipitation vs any species/colouration effects.

### Environmental variables

Three indices of environmental conditions (NAO, NDVI, and rainfall) were used to examine the relationships between feather characteristics and general annual wintering conditions. Finnish pied flycatcher wintering locations were estimated to be in West Africa between 5.5°N and 11.5°N, and 6.5°W and 15.5°W based on ring recoveries and geolocation data (n = 3 and n = 4, respectively) (Ouwehand et al. 2016). NDVI and rainfall values were calculated for terrestrial regions of the estimated wintering area as average values for February and March since pre-breeding moult peak of pied flycatchers occurs from mid-February to mid-March (Salewski et al. 2004). NAO (North Atlantic Oscillation index) values reflect climatic variation at a larger scale over terrestrial and marine areas in the Northern Hemisphere and winter NAO is routinely defined as average of values for December-March (Hurrell 1995; Jones et al. 1997). In Sub-Saharan and West Africa, negative NAO values correspond to wet winters and positive values to drier winters (Oba et al. 2001; Evan et al. 2006). NDVI (Normalized Difference Vegetation index) describes vegetation productivity of an area via remote sensing (Schmidt and Karnieli 2002). NDVI values range from of -1 to + 1 with higher values generally indicating greater productivity and more positive correlations with regional avian richness (Seto et al. 2004). The amount of rainfall was used as a third environmental index that directly reflects precipitation during the moulting period. NAO values were accessed at http://www.cru.uea.ac.uk/~timo/datapages/naoi.htm. Monthly means for NDVI and rainfall, available as 0.1° × 0.1° gridded rasters, were recorded by NASA Earth Observations (NEO) and were downloaded from http://neo.gsfc.nasa.gov.

### Isotope measurements

Feathers were soaked in a 2:1 chloroform:methanol solution overnight, rinsed, and air dried under a fume hood for 24 h. For stable-carbon and nitrogen isotope analyses, we weighed 1 mg of feather into precombusted tin capsules. Encapsulated feather was combusted at 1030 °C in a Carlo Erba NA1500 or Eurovector 3000 elemental analyser. The resulting N_2_ and CO_2_ were separated chromatographically and introduced to an Elementar Isoprime or a Nu Instruments Horizon isotope ratio mass spectrometer. We used two reference materials to normalize the results to VPDB and AIR: BWBIII keratin (*δ*^13^C = − 20.18, *δ*^15^N =  + 14.31‰, respectively) and PRCgel (*δ*^13^C = − 13.64‰, *δ*^15^N =  + 5.07‰, respectively). Within run (n = 5) precisions as determined from both reference and sample duplicate analyses were ± 0.1‰ for both *δ*^13^C and *δ*^15^N.

Samples for stable hydrogen (*δ*^2^H) isotopes were weighed (0.35 mg) into silver capsules using the feather barbs only. Capsules were compressed and analysed using the LSIS-AFAR stable isotope facility at the University of Western Ontario. Samples were loaded into a Uni-prep carousel (Eurovector®, Milan, ITA) held at 60ºC, evacuated and maintained under positive pressure with dry helium and then combusted in a Eurovector 3000 elemental analyzer pyrolytically on glassy carbon at 1350ºC. Separated H_2_ was analyzed using a Thermo Delta V Plus (Thermo scientific®, Bremen, DEU) continuous-flow isotope ratio mass spectrometer via a Conflo device (Thermo Scientific®, Bremen, DEU). Sample results were expressed in the standard delta (*δ*) notation in parts per thousand (‰) deviation from the Vienna Standard Mean Ocean Water (VSMOW) standard. In-house keratin standards (CBS: -197‰; KHS: -54.1‰) were used in order to derive the *δ*^2^H value of the non-exchangeable H fraction according to the comparative equilibration approach (Wassenaar and Hobson 2003). Based on within-run (n = 5 each) keratin standards, measurement error was estimated to be ± 2‰.

### Determining probable moult origins

**Fig. 1:**
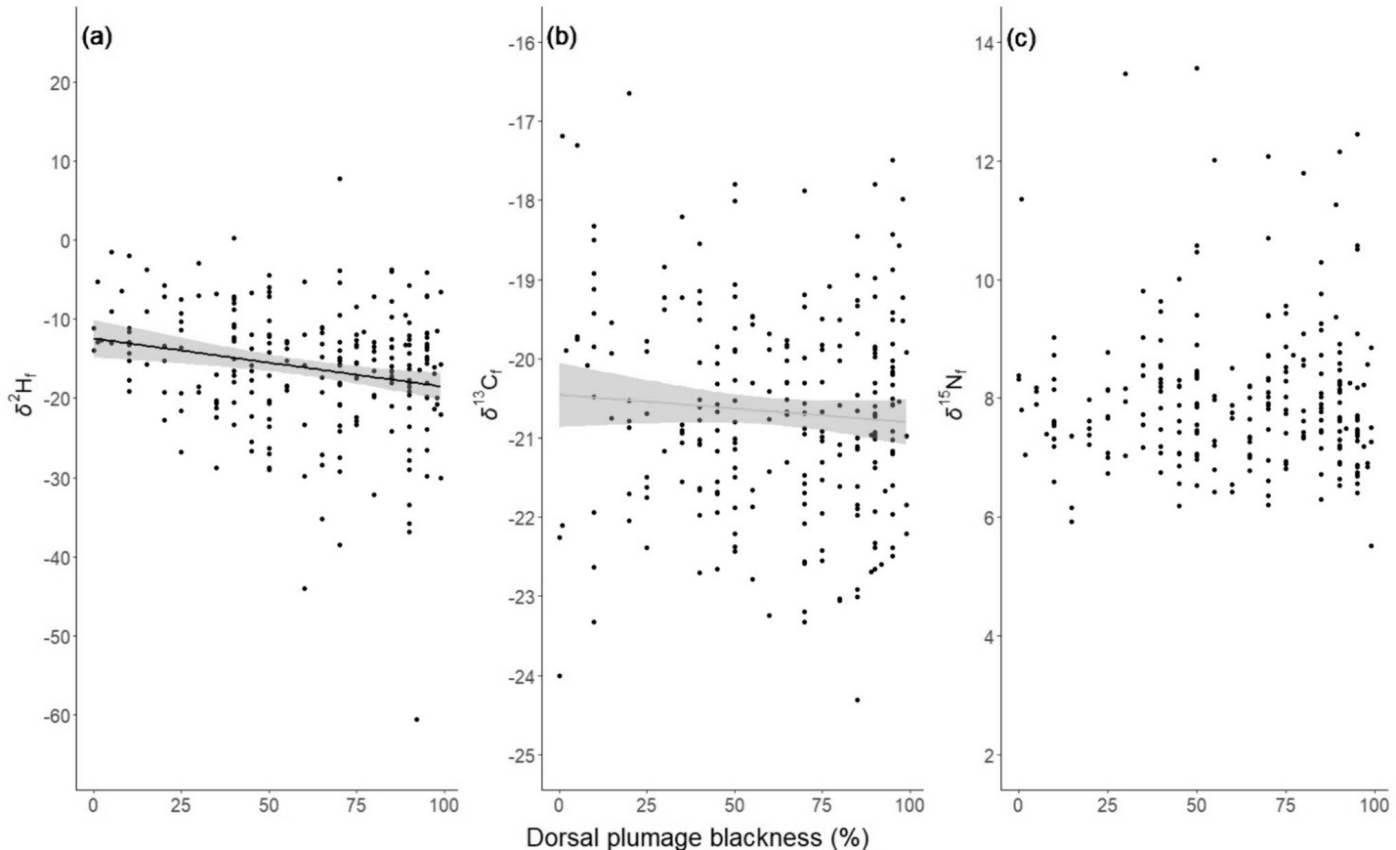
Associations between plumage blackness (in %) and feather isotope values (**a** hydrogen, **b** carbon, and **c** nitrogen in ‰) of individual pied flycatcher males. Significant association is indicated with black regression line, and non-significant (p = 0.08) tendency in **b** with grey regression line. In the analysis, dorsal plumage blackness is used as the response variable, but is presented here on the x-axis for illustration purposes

To assess if moult origins of male pied flycatchers varied with blackness, we used a dual-isotope multivariate normal probability density function (mvnpdf) method described in detail elsewhere (Hobson et al. 2014). In brief, we conducted probabilistic assignment to origin analyses using *δ*^2^H_f_ and *δ*^13^C_f_ restricted to possible moult origins in the western part of the pied flycatcher African non-breeding range. We excluded *δ*^15^N_f_ from the assignments due to difficulties in modelling this isotope spatially because of the likely influence of agricultural inputs. We conducted assignments separately for pied flycatchers with low (< 33%), moderate (33–66%) and high (> 66%) blackness values (Fig. 1 in Online Resource 2). The multi-isotope mvnpdf approach assumes that the isoscapes are independently governed by different biogeoclimatic processes and therefore exhibit spatial non-stationarity. We first converted an amount-weighted mean growing-season precipitation *δ*^2^H (*δ*^2^H_p_) isoscape surface (Bowen et al. 2005) to a feather isoscape using the calibration equation for known-origin migrant songbirds from Hobson et al. (2012a): *δ*^2^H_f_ = − 6.77 + 1.42* *δ*^2^H_p_. We used a *δ*^13^C isoscape representing the theoretical spatial distribution of *δ*^13^C values in plants in Africa, which is based on annual plant *δ*^13^C composition approximately corresponding to mean annual conditions (Still and Powell 2010) and applied + 2‰ to the *δ*^13^C isoscape to account for discrimination between plants and herbivorous insects in feather isotopes. We assumed that plant-based isoscapes exhibit minimal annual changes in *δ*^13^C and so this isoscape provided the most current and accurate approximation of plant *δ*^13^C composition available for Africa.

Following the mvnpdf analysis, we used a conservative odds ratio to assign feathers to potential moult origin using the spatially explicit probability densities for individual samples where georeferenced locations (i.e. raster cells) with ≥ 66.7% probability was coded as potential origins (1) and all other locations (i.e. < 66.7%) were considered as improbable origins (0). Assignment to origin analyses conducted for each sample resulted in a spatially referenced binary raster file for each individual, which were subsequently summed across assignments for all individuals to represent potential origins in each blackness grouping. Assignment to origin analyses including spatial file manipulation were conducted using the ‘rgeos’, ‘mvtnorm’,’sf’, ‘sp’ and ‘Rfast’ packages in the R v4.1.1 computing environment (Genz and Bretz 2009; Bivand et al. 2013; Pebesma 2018; Bivand and Rundel 2023; Papadakis et al. 2023; Pebesma and Bivand 2023).

### Statistical analyses

**Fig. 2:**
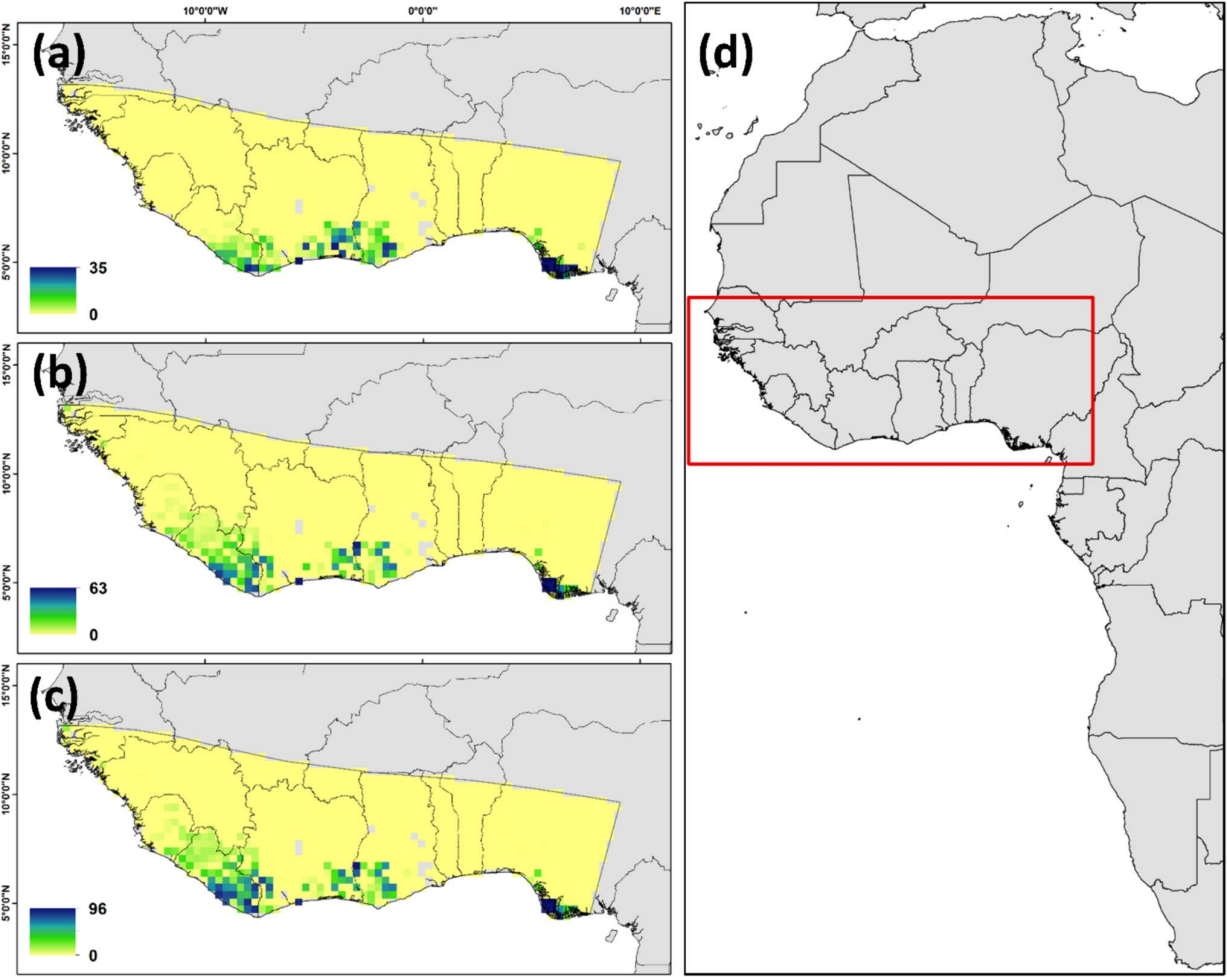
Depictions of purported non-breeding moult origins of male pied flycatchers with different blackness values (**a** < 33% blackness, N = 40; **b** 33–66% blackness, N = 77; **c** > 66% blackness, N = 119) assigned to the western part of their African non-breeding range **d** based on similarity in isotope values to the underlying isoscapes using a multivariate probability density function (see “Materials and methods”). Values in the legend indicate the minimum and maximum number of individuals potentially originating from a particular cell in the dual *δ* ^2^H_f_ and *δ* ^13^C_f_ isoscape

First, the relationships between plumage blackness and each stable isotope value were tested with a linear mixed model using percent blackness as the response variable and *δ*^13^C_f_, *δ*^15^N_f_, or *δ*^2^H_f_ values as individual explanatory variables. Age (young or old) was also included in the model as a fixed effect to control for possible age effects, as male pied flycatcher plumage tends to slightly darken (ca. 10–15%) between the ages 1 and 2 years (Lundberg and Alatalo 1992). Individual ring number was included as a random effect as some individuals (n = 31) were measured more than once. In this dataset, the year of feather collection by default did not explain any variance in plumage blackness, as individuals from each year were selected to represent a similar continuum from brown to black plumage (Fig. 2 in Online resource 2). Thus, year was not included in this analysis. Links between feather isotope values and the environmental variables were also explored to connect yearly local wintering conditions to general annual wintering conditions (Online resource 1).

**Table 1:** Results of the linear mixed model explaining the variation in plumage blackness in relation to feather isotopes (hydrogen = δ^2^H_f_, carbon = δ^13^C_f_, and nitrogen = δ^15^N_f_) and age (young or old)

Independent variable	Plumage blackness	df	t	p
	Estimate ± 1se			
Fixed effects				
Intercept	10.24 ± 27.83	219.43	0.37	0.71
** Hydrogen**	**− 0.50 ± 0.21**	**211.50**	**− 2.33**	**0.02**
Carbon	− 2.51 ± 1.41	224.96	− 1.78	0.08
Nitrogen	− 0.30 ± 1.49	222.52	− 0.20	0.84
** Age (young)**	**− 19.30 ± 3.61**	**220.03**	**− 5.34**	** < 0.001**

Random effects	Variance ± 1sd			
Ring	365.80 ± 19.12			
Residual	314.80 ± 17.74			

Bolded variables indicate significant effect

Second, we modelled the local return probability of male pied flycatchers as a function of their colouration, local wintering conditions (feather isotope values) and general wintering conditions (environmental indices for the winter prior to return). Mixed effects Cox regressions were used for this analysis because of repeated individual measurements in the data and performed with functions ‘coxme’ and ‘Surv’ from the packages ‘coxme’ and ‘survival’ (Therneau 2022, 2023). Separate models were run for each isotope × environmental variable interactions (9 models) against the binary response variable (returned or not) including age at first capture (young or old) as a fixed effect. Individual identification was used as a random effect. Similar models were run for interactions between plumage coloration and environmental variables (3 models).

Our study suffers from an inherent limitation of not being able to link the wintering habitat of a specific year to the return rate of the same year as we lacked relevant feathers, from which to measure the isotope values from those individuals that did not return. However, Salewski et al. (2000) reported previously that almost a quarter (23.4%) of pied flycatchers that were captured in one wintering site in Africa returned to the exact same site in the following years. Thus, we used our stable isotope values as a proxy for conditions experienced by the individual also in the coming years. This approach assumes that the isotope values are repeatable within individuals. Thus, to determine whether birds returned to similar winter sites in different years, within-individual repeatabilities were calculated with each isotope as a response variable and individual ring number as a random effect using the function ‘rpt’ from the package ‘rptR’ (Stoffel et al. 2017).

All linear mixed models were run using the function ‘lmer’ in the package ‘lme4’ (Bates et al. 2015) and estimated using the restricted maximum likelihood method. Statistical significances for explanatory variables were obtained using the package ‘lmerTest’ (Kuznetsova et al. 2017). Degrees of freedom for fixed factors were calculated and parameter estimates, and their standard errors were assessed using the Kenward-Roger method with the package ‘pbkrtest’ (Halekoh and Højsgaard 2014). Statistical significance (α) was set at 0.05. All statistical analyses were conducted with R version 4.1.2 (R Core Team 2023).

## Results

**Fig. 3:**
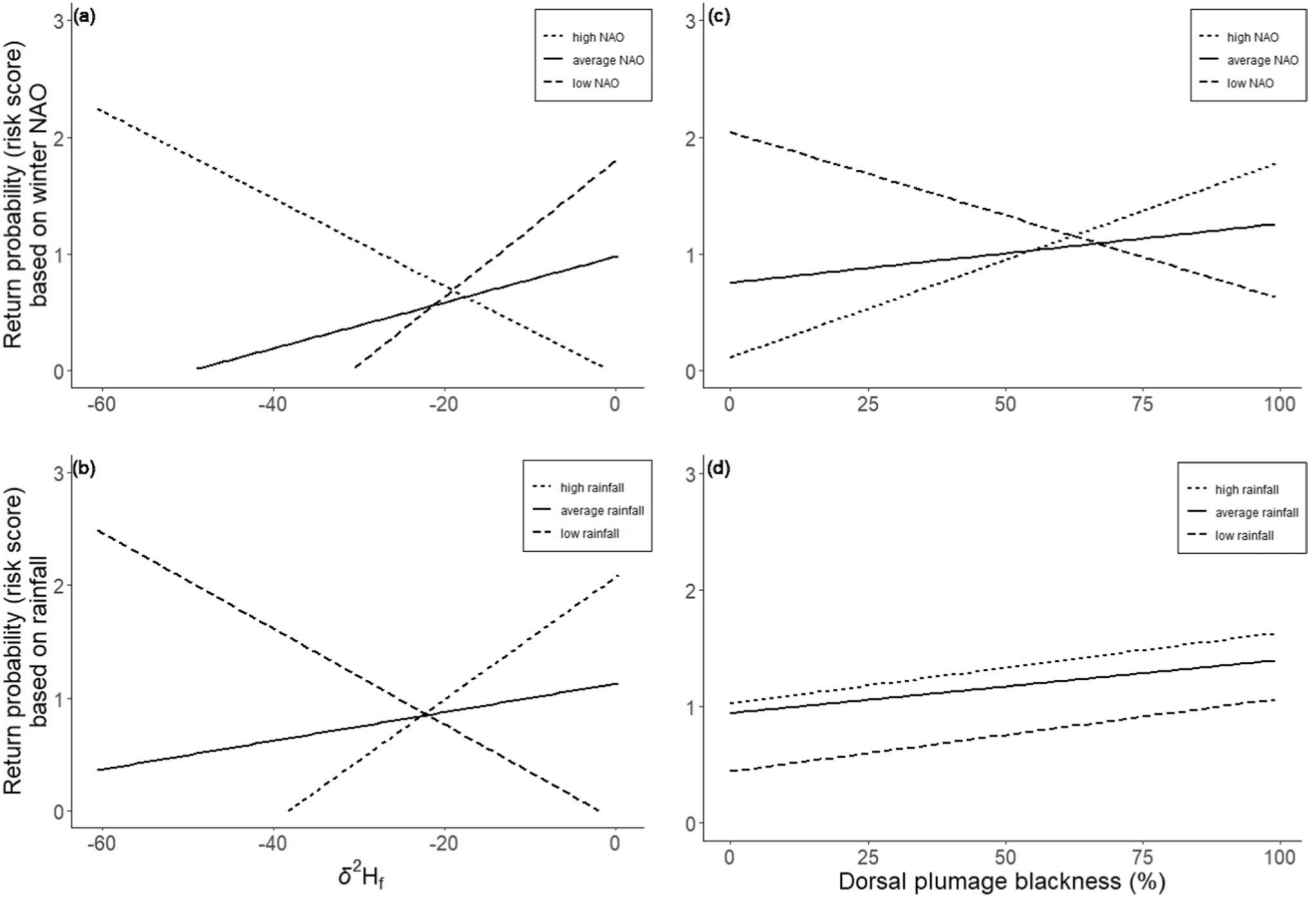
Male pied flycatcher probability of return as model predicted risk scores (> 1 = increased probability to return; < 1 = decreased probability to return) in the next breeding season as a function of feather hydrogen isotope values (left side figures) or plumage blackness in % (right side figures) for winters with different NAO indices (upper row) or monthly average rainfall (lower row) during feather formation. Model predictor lines for high NAO/Rainfall values are indicated by dotted line (2.08/68.5 mm), for average values by solid line (− 0.31/57.1 mm), and for low values by dashed line (− 2.71/41.1 mm). These values are 10%, 50%, and 90% quantiles of the data. Environmental indices are shown as categorical for illustration but were treated as continuous variables in the analyses

Both *δ*^13^C_f_ and *δ*^15^N_f_ values were highly repeatable within individuals across years (C: 0.685 (95% CI [0.50, 0.82], P < 0.001); N: 0.804 (95% CI [0.68, 0.90], P < 0.001)). In contrast, *δ*^2^H_f_ values were not repeatable (H: 0.0 (95% Cl [0, 0.38], p = 0.5)) (Fig. 3 in Online resource 2), which was expected, as *δ*^2^H_f_ values are linked with rainfall that varies among years. Local between-year changes in rainfall amount occur and likely result in low repeatability in *δ*^2^H_f_ values, while differences in average rainfall across multiple regions are less likely to change (Mohr 2004). Furthermore, male plumage colour was repeatable within individuals across years (R = 0.7 (95% CI [0.64, 0.76], P < 0.0001) in a larger sample of this population as reported in Järvistö et al. (2016), and R = 0.59 (95% CI [0.34, 0.78], P = 0.001) in these data for the 31 repeated individuals).

Male pied flycatcher plumage blackness was negatively related with *δ*^2^H_f_ values but not significantly related with *δ*^13^C_f_ or *δ*^15^N_f_ values (Table 1, Fig. 1). Browner males had higher *δ*^2^H_f_ values while blacker males exhibited, on average, lower *δ*^2^H_f_ values indicative of mesic habitats (Fig. 1a). To examine the variation of *δ*^2^H_f_ values across the colouration gradient we ran a separate model regressing *δ*^2^H_f_ values against the blackness index and regressed the residual absolute values of this model again against blackness. According to this analysis, there was non-significant (p = 0.15), positive relationship between blackness and variation in *δ*^2^H_f_ values (Fig. 4 in Online resource 2). Male pied flycatchers appeared to originate (i.e. had isotopic profiles that aligned with the underlying isoscapes) from generally similar regions in the southern part of their non-breeding range from Liberia to Nigeria regardless of blackness (Fig. 2, Fig. 1 in Online resource 2). However, assignment analyses showed a potential regional distinction between moulting sites of browner and blacker males; individuals with blackness values < 33% seem to have moulted in areas concentrated in eastern Liberia, southern Ivory Coast, western Ghana and southern Nigeria whereas individuals with blackness values > 33% had potential origins in more western areas across Liberia in addition to the regions of origin similar to birds with < 33% blackness (Fig. 2).

Across years, low *δ*^2^H_f_ values, but not *δ*^13^C_f_ nor *δ*^15^N_f_ values were linked to higher precipitation winters (Online resource 1, and Fig. 5 in Online resource 2). Values of *δ*^2^H_f_, indicative of different local environmental conditions, were associated with plumage blackness in the cross-sectional sample and so we tested whether a change in winter conditions influenced the colour change in breeding plumage between years at the individual level. Using individuals with repeated samples, the between-year change in plumage colour was tested against the between-year change in *δ*^2^H_f_ value while controlling for age, but no relationship was found (β = 0.21, se = 0.45, t = 0.46, p = 0.65).

Values of *δ*^13^C_f_ and *δ*^15^N_f_ or their interactions with environmental indices did not influence the return probability of male pied flycatchers to the breeding grounds (Table 2). However, *δ*^2^H_f_ values had a significant interaction with both NAO (Table 2, Fig. 3a) and rainfall (Table 2, Fig. 3b) where individuals with low *δ*^2^H_f_ values were more likely to return when NAO was higher, and rainfall was lower the following winter (i.e. winter prior to their return). Despite the lack of within-individual repeatability in *δ*^2^H_f_ values as reported above, which hampers the use of single *δ*^2^H_f_ measurements to infer future measures, plumage colour is repeatable within individuals. Consequently, as *δ*^2^H_f_ values were negatively associated with plumage blackness, a similar trend with return rate was found for the interaction between plumage coloration and NAO (Blackness × NAO: β = 0.007, se = 0.004, z = 1.88, p = 0.06, Fig. 3c) but not for plumage colouration and rainfall (Fig. 3d). Therefore, blacker individuals tended to return at a higher rate than browner individuals after high NAO winters, while browner individuals were more likely to return after low NAO winters. However, as we were unable to provide direct evidence on the association between *δ*^2^H_f_ values and return rate, we consider this evidence as circumstantial.

**Table 2:** Interaction (isotope × environmental index) results from mixed effects Cox regressions modelling return probability of male pied flycatchers to Finnish breeding grounds as a function of local wintering conditions (as represented by feather isotopes) and annual general wintering conditions (environmental indices)

Interaction	Probability to return		
	Estimate ± se	z	p
**δ**^**2**^**H**_**f**_** × NAO**	**− 0.04 ± 0.01**	**− 3.15**	**0.002**
δ^2^H_f_ × NDVI	2.02 ± 1.26	1.61	0.11
**δ**^**2**^**H**_**f**_** × Rain**	**0.004 ± 0.002**	**2.64**	**0.008**
δ^13^C_f_ × NAO	0.08 ± 0.08	0.98	0.33
δ^13^C_f_ × NDVI	− 5.73 ± 7.26	− 0.79	0.43
δ^13^C_f_ × Rain	− 0.02 ± 0.01	− 1.60	0.11
δ^15^N_f_ × NAO	0.05 ± 0.09	0.55	0.58
δ^15^N_f_ × NDVI	5.88 ± 8.27	0.71	0.48
δ^15^N_f_ × Rain	0.01 ± 0.01	0.85	0.39

The models included the main effects, individual identity as a random effect, and controlled for age (young vs. old) at first capture (in all models p > 0.1). Bolded variables indicate significant effect

## Discussion

As predicted, feather *δ*^2^H values, but not *δ*^13^C or *δ*^15^N values, were associated with plumage blackness in male pied flycatchers so that, on average, *δ*^2^H_f_ values declined with increasing plumage blackness. The individual change in *δ*^2^H_f_ values did not explain the change in plumage blackness across years, indicating that differently coloured males inhabited different environments rather than indicating that environment influenced plumage colouration. Similarly, consistent with Ouwehand et al. (2016), we found high repeatability of both *δ*^13^C_f_ and *δ*^15^N_f_ values suggesting that individuals tend to return to the same, or at least similar isotopic areas or habitats for the winter year after year. As expected, males with lower *δ*^2^H_f_ values were more likely to return to the same breeding site than males with higher *δ*^2^H_f_ values after dry winters but the effect was reversed when winters were wetter.

### Associations between plumage colour and habitat

In Africa, pied flycatchers inhabit broadleaved forests, shrublands, and grasslands (BirdLife International 2022) that vary in rainfall (Salewski et al. 2002b). Higher feather *δ*^2^H values have previously been associated with lower regional rainfall (Hobson and Wassenaar 1996; Hobson et al. 2012b) consistent with the rainfall “amount effect” describing local precipitation *δ*^2^H (Clark and Fritz 1997). Plumage and feather characteristics are often influenced by habitat and conditions during feather formation (e.g., Saino et al. 2004; Eggers and Low 2014; Meillère et al. 2017), and also pied flycatcher males have been shown to moult into darker plumage after drier winters (Järvistö et al. 2016). Here, we however show that individual change in plumage colouration did not follow the change in *δ*^2^H values indicating that local rainfall does not affect plumage coloration. This suggests that browner males tend to winter mainly in drier habitats while blacker males tend to be found in wetter areas. As there were no significant relationships between plumage coloration and feather *δ*^13^C or *δ*^15^N values, it appears that pied flycatchers overwinter in otherwise isotopically similar areas but that differ in the amount of annual precipitation. However, there was a tendency for *δ*^13^C_f_ values to decline with increasing plumage darkness, also indicating that browner individuals overwinter in drier habitats. Plants with a C3 photosynthetic pathway respond to heat and water stress by reducing stomatal openings thereby increasing their *δ*^13^C values. Our data underline the fact that local overwinter habitats consisted of C3 and C4 plants that represent long-term climatic averages (where food webs remain relatively constant in average *δ*^13^C) whereas rainfall amount is expected to be more variable among years and more directly linked to year-specific food web *δ*^2^H values (but see Vander Zanden et al. 2015). The broad likely wintering regions recognised by the assignment analysis align with wintering areas of the pied flycatcher identified using light-level geolocators, which showed moderate within-population connectivity (Ouwehand et al. 2016). While we cannot rule out that individuals with different blackness values overwinter in different regions based on this analysis, it is equally possible that these individuals use different habitats as described above.

Although wintering pied flycatcher males seem to distribute according to Gloger’s rule (Gloger 1833), the rule likely cannot be applied to the overwinter period because all pied flycatcher males are brown on their wintering grounds and only become darker after moult shortly before spring migration (Lundberg and Alatalo 1992; Svensson 1992). Instead, differences in individual competitive ability might influence the capacity to occupy and moult in different quality habitats in winter (Salewski et al. 2002b; Reudink et al. 2009). As insect abundance increases with rainfall (Sinclair 1978; Studds and Marra 2007), wetter habitats in Sub-Saharan Africa are arguably better habitat than drier areas for insectivorous passerines (López-Calderón et al. 2017). Previous research showed that pied flycatchers hold wintering site territories (Salewski et al. 2002a) and good competitive ability helps them acquire a good territory with more rainfall. The lowest *δ*^2^H_f_ values reflecting a wetter territory were found only in darker male pied flycatchers. Generally, darker melanin-based coloration is linked to aggressive behaviour through pleiotropic effects of the genes responsible for melanin production (Ducrest et al. 2008). Indeed, at the breeding grounds, darker males exhibit more territorial activity than lighter males (Slagsvold and Lifjeld 1988). Early evidence suggests that they also acquire better, more deciduous breeding territories than lighter males (Järvi et al. 1987). However, later studies may have obscured the potential link between plumage colour and breeding territory quality, potentially by offering a surplus of good nesting territories (Lundberg and Alatalo 1992; Silverin 1998) and links between plumage colour, aggressiveness, and competitive ability in this species have been similarly inconsistent (Järvi et al. 1987; Breiehagen and Sætre 1992; Huhta and Alatalo 1993).

The reproductive success of differently coloured pied flycatcher males depends on weather during different stages of breeding (Sirkiä et al. 2010; Järvistö et al. 2015), suggesting that differently coloured pied flycatchers could be adapted to different conditions, at least during the breeding season. Within-species adaptations to distinctive non-breeding habitats have been reported with other bird species, but these differences were attributed to morphology-induced differences in foraging strategy (Satgé et al. 2022) and personality (Chyb et al. 2021) rather than phenotypic differences. However, it is possible that differently coloured pied flycatchers occupy different wintering habitats because they favour different environmental conditions (Galeotti and Rubolini 2003; Roulin 2004; Forsman and Åberg 2008), potentially through the pleiotropic effects of melanin production and/or other genetic correlations (Ducrest et al. 2008; McKinnon and Pierotti 2010).

### Climate, plumage and return rate

Individuals that wintered in areas with more rainfall (as indicated by lower feather *δ*^2^H values) were more likely to return to the same breeding site when the winter prior to returning was overall drier than average. While NAO represents a general global climatic index, different areas vary in mean rainfall within years (Jones et al. 1997; Mohr 2004). Therefore, in high NAO years, areas with higher-than-average rainfall more likely still receive some rain while other areas might suffer from drought. Interestingly, the return probability of individuals that wintered in wetter areas decreased with decreasing NAO and increasing rainfall. While sufficient rain likely increases survival over the non-breeding season and/or contributes to achieving good conditions for return migration (Marra et al. 1998; Rockwell et al. 2017), heavy rainfall can decrease the activity of aerial insects leading to reduction in feeding opportunities of insectivores (Veistola et al. 1997; Cowley and Siriwardena 2005) which consequently could lead to lowered individual body condition and insufficient preparation for migration.

As browner individuals winter more in drier habitats compared to blacker individuals, the weak positive connection between plumage blackness and return rate after high NAO winters was not surprising. Using a larger dataset from the whole population, Järvistö et al. (2016) found that the proportion of blacker individuals in the Ruissalo breeding population increased after high NAO winters corresponding to drier conditions. Overall dry winters likely benefit blacker males occupying locally wetter areas as poor wintering conditions can hinder fuelling for migration which may delay migration departure and subsequently lead to later spring arrival date (Marra et al. 1998; Gunnarsson et al. 2006; Ouwehand and Both 2017). In the case of a nest box population of pied flycatchers, this could result in late males settling outside the study area in often poor quality natural cavities (Lundberg and Alatalo 1992), which would leave them out of the breeding population monitoring. When environmental conditions in northern Central Europe are favourable during northward migration, the proportion of browner males in our breeding population increased, indicating a prolonged migration of Central European brown male pied flycatchers (Sirkiä et al. 2013). Conversely, unfavourable environmental or poor individual conditions might shorten the spring migration of browner individuals so that they do not return to the previous, more northern breeding sites, but this hypothesis remains to be studied.

Our study revealed an association between inferred habitat quality on African wintering grounds and the return rate of differently coloured male pied flycatchers to Finland. This contrasts with the previously held notion that breeding plumage colour is not related to overwinter survival, as indicated by return rates (Lundberg and Alatalo 1992). Return rates of pied flycatcher males were investigated in the 1980s and early 1990s in Scandinavia and Spain, where males were classified as black or brown, and depending on the year, either higher return rates of brown or black males, or no differences between the classes were found (Røskaft et al. 1986; Slagsvold and Lifjeld 1988; Potti and Montalvo 1991; Alatalo et al. 1994). Interestingly, winter NAO values across these study periods varied greatly (1981–91 values from -0.38 to 2.86, average of 0.85). Especially high NAO values preceded the breeding seasons in 1983 and 1989 (NAO = 2.00 and 2.86, respectively, cf. 2.08, the highest NAO value within current study period), when higher return rates of black males to the breeding grounds were also observed, similar to our study. Male plumage colouration has strong effects on the breeding success of the breeding pair, but in a temperature related manner where nestling mortality of blacker males is higher than browner males when it is cold (Sirkiä et al. 2010), and blacker males produce heavier fledglings when it is warm during the nestling period, but lighter fledglings than those of browner males when it is cold (Järvistö et al. 2015). Thus, mismatch between wintering and breeding conditions (i.e. wintering conditions favouring one colour morph but breeding conditions the other) would likely have dire consequences for the breeding success of the population. As far as we know, no study has investigated direct links between wintering conditions and subsequent breeding success in differently coloured male pied flycatchers. Teerikorpi et al. (2018) found that after high NAO, thus drier winters, male pied flycatchers with larger white wing patches attracted females that laid larger clutches which also had better local survival to the following breeding seasons, while the effect was reversed after low NAO winters. White wing patches have been previously shown to get smaller during high NAO winters, while large wing-patched males had higher return rates to the breeding population than small wing-patched males after drier winters. This pattern was reversed after moister winters (Järvistö et al. 2016), similarly as in the current study in relation to winter NAO and black plumage colouration. These results together elaborate the influence of wintering conditions on phenotypic compositions of breeding populations, and ultimately breeding success through return rates and male quality in the breeding environment.

## Conclusions

Global climate change is altering local environments experienced by different species. Genetic diversity, as reflected in degree of colour polymorphism, can increase the resilience of a species against climate change, but one morph might be favoured over others (Roulin 2014). Indeed, observed changes in phenotypic abundance within populations can indicate environmental changes over large temporal scales (Karell et al. 2011). Stable isotope measurements in feathers are an important proxy for winter habitat use on the moulting grounds. Across Europe, pied flycatcher populations with mostly brown males have experienced more dramatic declines in past decades than populations with both browner and blacker males (Both et al. 2006; Lehikoinen and Piha 2021; Nater et al. 2022). Using a breeding population consisting of male pied flycatchers of both colour extremes, our isotope study combined with NAO index showed that browner males winter in drier areas more than blacker males, and have reduced return rates after overall drier winters. If this pattern also holds true in other populations, the brown morph of the pied flycatcher may be at risk of declining as melanin-coloration is heritable, and according to climate change projections, drier parts of West Africa are expected to get even drier, while the moist eastern parts are expected to experience more, and heavier, rainfall (Trisos et al. 2022). Morph-specific population declines reduce the genetic diversity of a species, rendering it less resilient against further changes in climatic conditions. Future studies should therefore investigate the wintering conditions of other brown males, but also of females, which are always brown and fundamentally important for the resilience of a species. Changing conditions likely affect not only pied flycatchers but other Afrotropical migrant species wintering in the same areas. In addition to changing climate, alteration of wintering habitats due to increased deforestation related to intensified agriculture and grazing, are important drivers of declining breeding population trends of European migrants wintering in West Africa (Howard et al. 2020). Effective conservation of long-distance migrant birds, such as the pied flycatcher, thus would require targeted land management actions in Africa that ensure conservation of suitable wintering areas while considering of future climate change scenarios.

### Supplementary Information

Below is the link to the electronic supplementary material.

Supplementary file 1 (PDF 572 KB)

Supplementary file 2 (PDF 774 KB)

## Data Availability

Data used in this study are available in Figshare (10.6084/m9.figshare.25674441).
