# Supplementary material for: Winter-ground microhabitat use by differently coloured phenotypes affects return rate in a long-distance migratory bird
Source: Oecologia. 2024 May 9;205(1):163–76. doi: 10.1007/s00442-024-05561-8 (PMC11144160; doi:10.1007/s00442-024-05561-8)
Supplement: Supplementary file 1 — (PDF 572 KB) [file 442_2024_5561_MOESM1_ESM.pdf]

Electronic Supplemental Material – ESM1

Winter-ground microhabitat use by differently coloured phenotypes affects return rate in a long-distance migratory bird

Tiia Kärkkäinen[1,2]*, Keith A. Hobson[3,4], Kevin J. Kardynal[4], Toni Laaksonen[1]

[1]Department of Biology, University of Turku, Turku, Finland
[2]Department of Evolutionary Ecology, National Museum of Natural Sciences, Madrid, Spain
[3]University of Western Ontario, London, Canada
[4]Environment and Climate Change Canada, Saskatoon, Canada
*Corresponding author: tmakark@gmail.com

Connecting local wintering conditions with year-specific conditions on the species' general African wintering area

Stable isotopes are known to enrich in animal and plant tissues in dry conditions, thus in general, rainier weather is associated with lower isotope values (Clark & Fritz, 1997; Hobson et al., 2012). We explored this connection in our data by using measured isotope values as indicators of local conditions (C, N, H) and large-scale environmental indices calculated for the estimated general wintering area of pied flycatchers as indicators of general conditions (NAO, NDVI, rainfall). Based on the literature, we expected that in overall dry years, isotope values would show enrichment, and in overall wet years, isotope values would be lower.

Methods

All environmental indices were separately mean-centered by subtracting the group mean (mean of the yearly values of 2007-2014) from each individual year value. All environmental variables were strongly correlated (NAO – NDVI = -0.49; NAO – Rainfall = -0.78; NDVI – Rainfall = 0.82), so to avoid multicollinearity issues and reduce dimensionality in these variables, a principal component regression was performed using function 'prcomp' in the package 'stats' (R Core Team, 2023) to combine correlated environmental variables across years into independent principal components, and to obtain year-specific PC values. Variable contributions to the principal components were extracted with function 'get_pca_var' in the package 'factoextra' (Kassambara & Mundt, 2020). Thereafter, three different linear mixed models were run with an isotope value (H, C, or N) as the response variable, PC1 as the explanatory variable and year as a random effect. As environmental variables in these data differed only among years, the sample size for the environmental variables was low (n = 8 years). Therefore, to confirm results of the principal component regression, each environmental variable was run alone against each isotope-value, keeping year as a random effect.

Results

The first principal component (PC1) of the PCA explained 80% of the variation in the three environmental variables (NAO, NDVI, and rain) across years. All three variables contributed similarly to the first principal component (NAO 30%, NDVI 31%, and rain 39%). NAO was negatively associated with PC1, while both NDVI and rain were positively associated with PC1 (Fig.1). Thus, high PC1 value indicates low NAO (wet winters), high NDVI (increased primary production), and high rainfall. PC1 was negatively associated with δ^2H_f (β = -1.16, se = 0.33, $t_{6.0}$ = -3.5, p = 0.01, Fig.3A), but not with other isotopes ($\delta^{13}C_f$: β = 0.05, se = 0.07, $t_{6.0}$ = 0.7, p = 0.49, Fig.3B; $\delta^{15}N_f$: β = -0.04, se = 0.07, $t_{6.0}$ = -0.6, p = 0.60, Fig.3C). Therefore, winters with above average precipitation and moisture were characterized by lower δ^2H_f values, but they did not differ in $\delta^{13}C_f$ of $\delta^{15}N_f$ values compared to drier winters. This result was repeated with models using a single environmental factor as an explanatory variable: NAO had a positive relationship (β = 1.19, se = 0.32, $t_{5.9}$ = 3.77, p = 0.01) and

rainfall a negative relationship (β = -0.18, se = 0.05, $t_{6.0}$ = -3.44, p = 0.014) with δ^2H_f values, but not with other isotopes. However, NDVI had no relationship with any isotope in these models.

References:

Clark, I. D., & Fritz, P. (1997). Environmental Isotopes in Hydrogeology. CRC Press.

Hobson, K. A., Wilgenburg, S. L. V., Wassenaar, L. I., & Larson, K. (2012). Linking Hydrogen (δ2H) Isotopes in Feathers and Precipitation: Sources of Variance and Consequences for Assignment to Isoscapes. PLOS ONE, 7(4), e35137. https://doi.org/10.1371/journal.pone.0035137

Kassambara, A., & Mundt, F. (2020). factoextra: Extract and Visualize the Results of Multivariate Data Analyses. R package version 1.0.7, https://CRAN.R-project.org/package=factoextra

R Core Team. (2023). R: A language and environment for statistical computing [Computer software]. R Foundation for Statistical Computing. http://www.R-project.org

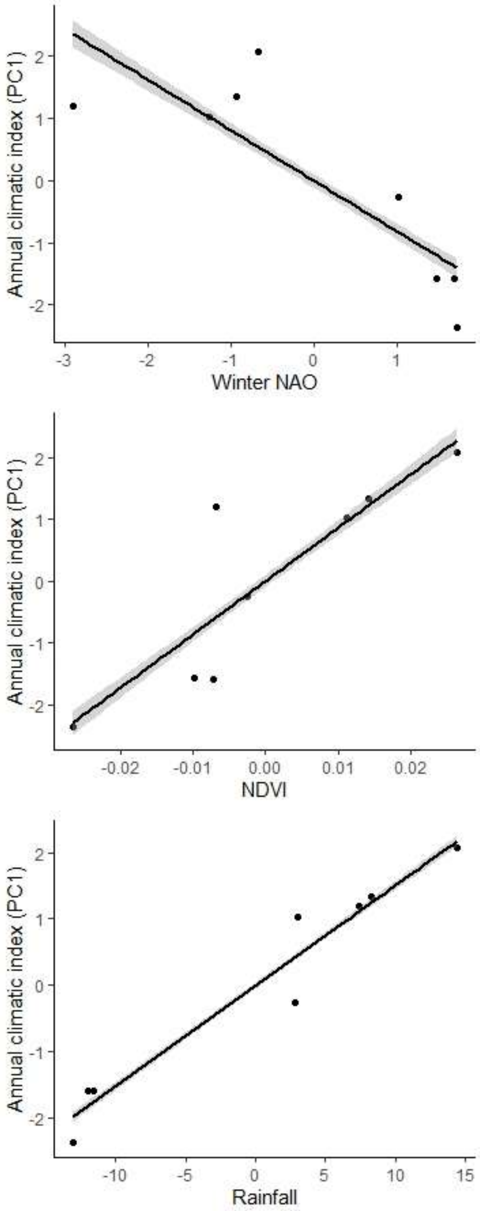

ESM1, Figure 1. Associations between PC1 and the three climatic variables.
