# Supplementary material for: Winter-ground microhabitat use by differently coloured phenotypes affects return rate in a long-distance migratory bird
Source: Oecologia. 2024 May 9;205(1):163–76. doi: 10.1007/s00442-024-05561-8 (PMC11144160; doi:10.1007/s00442-024-05561-8)
Supplement: Supplementary file 2 — (PDF 774 KB) [file 442_2024_5561_MOESM2_ESM.pdf]

Electronic Supplemental Material – ESM2

Winter-ground microhabitat use by differently coloured phenotypes affects return rate in a long-distance migratory bird

Tiia Kärkkäinen[1,2]*, Keith A. Hobson[3,4], Kevin J. Kardynal[4], Toni Laaksonen[1]

[1]Department of Biology, University of Turku, Turku, Finland
[2]Department of Evolutionary Ecology, National Museum of Natural Sciences, Madrid, Spain
[3]University of Western Ontario, London, Canada
[4]Environment and Climate Change Canada, Saskatoon, Canada
*Corresponding author: tmakark@gmail.com

Other supporting figures

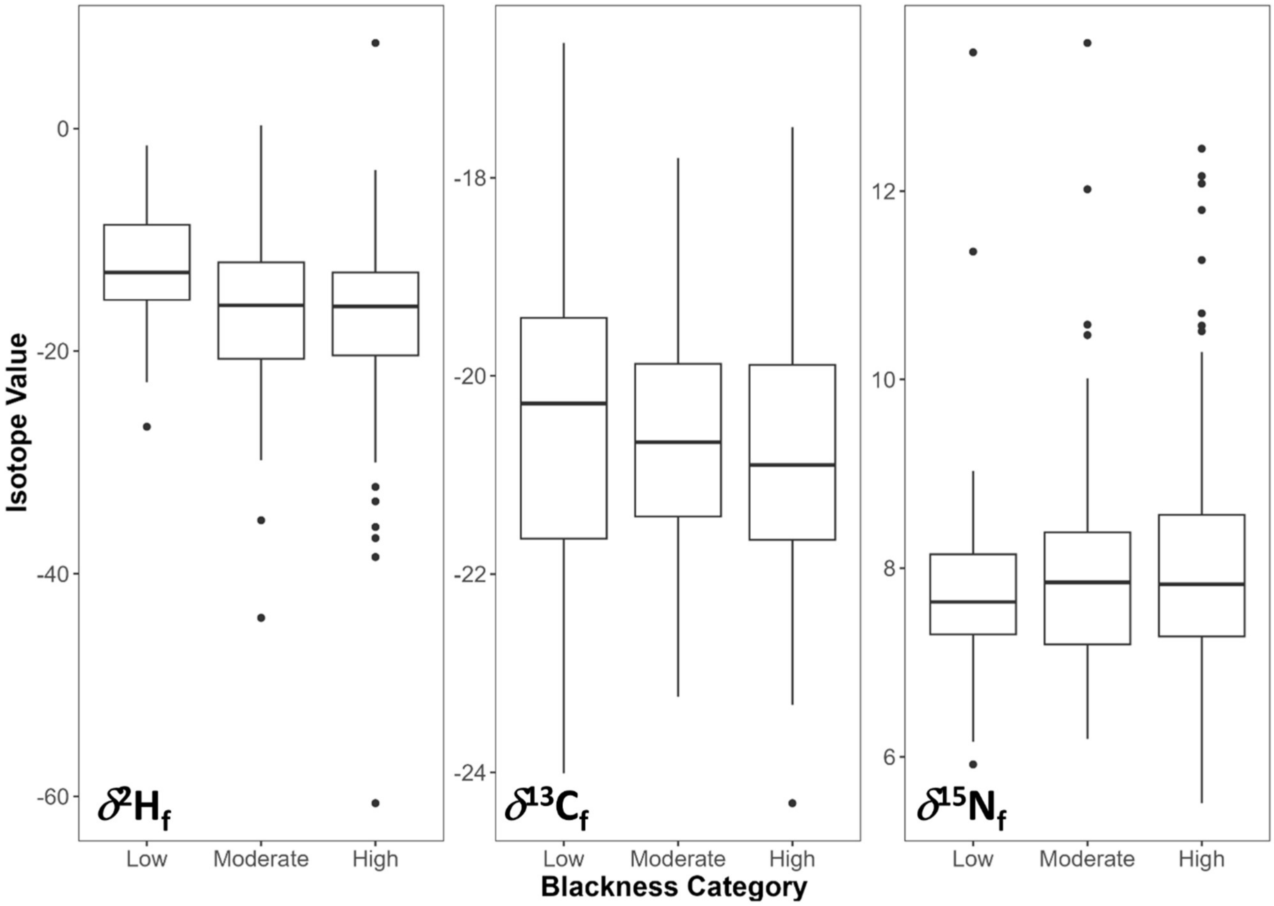

ESM2, Figure 1. Boxplots showing variation in feather isotope (δ^2H_f, $\delta^{13}C_f$, $\delta^{15}N_f$) values by blackness category in male pied flyatchers (Low: <33% blackness; Moderate: 33 – 66% blackness; High: >66% blackness).

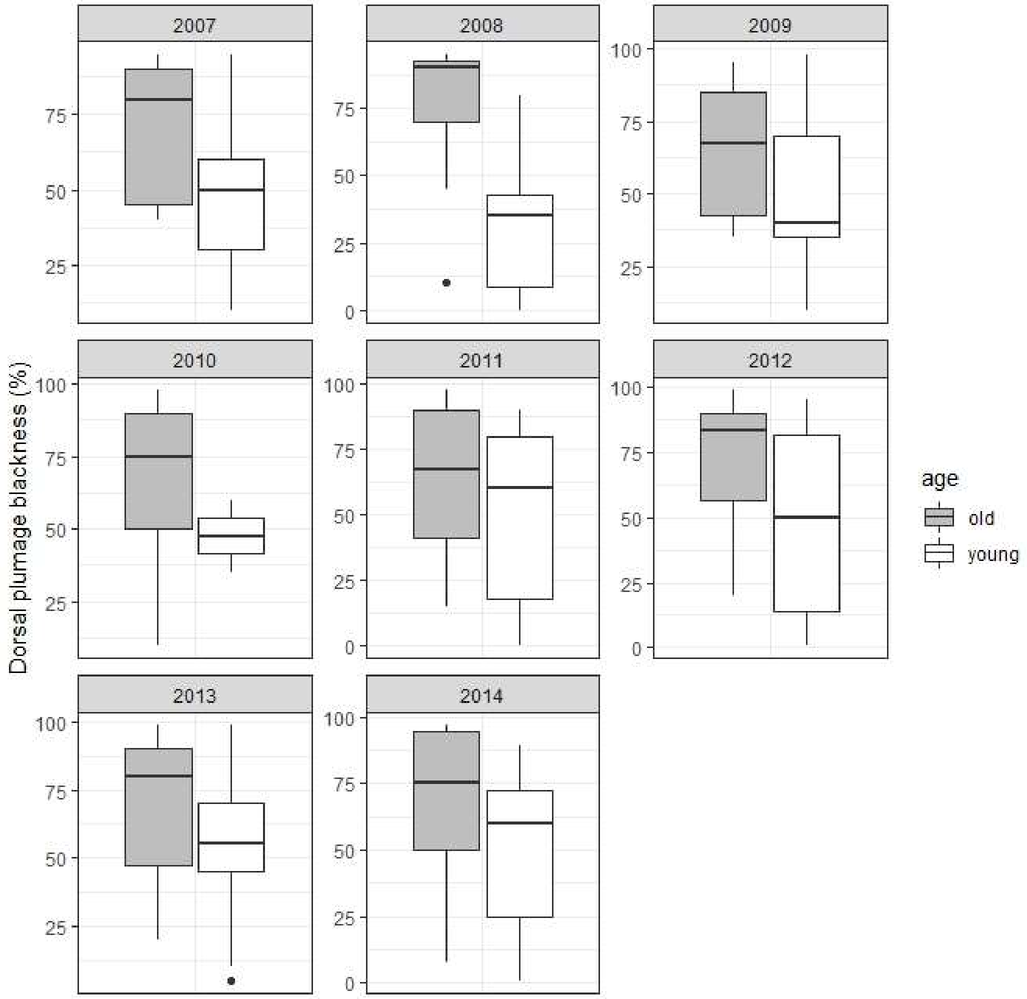

ESM2, Figure 2. Boxplots indicating annual variation in dorsal plumage blackness by age of male pied flycatchers (young = 1 year old, old = 2+ year old)

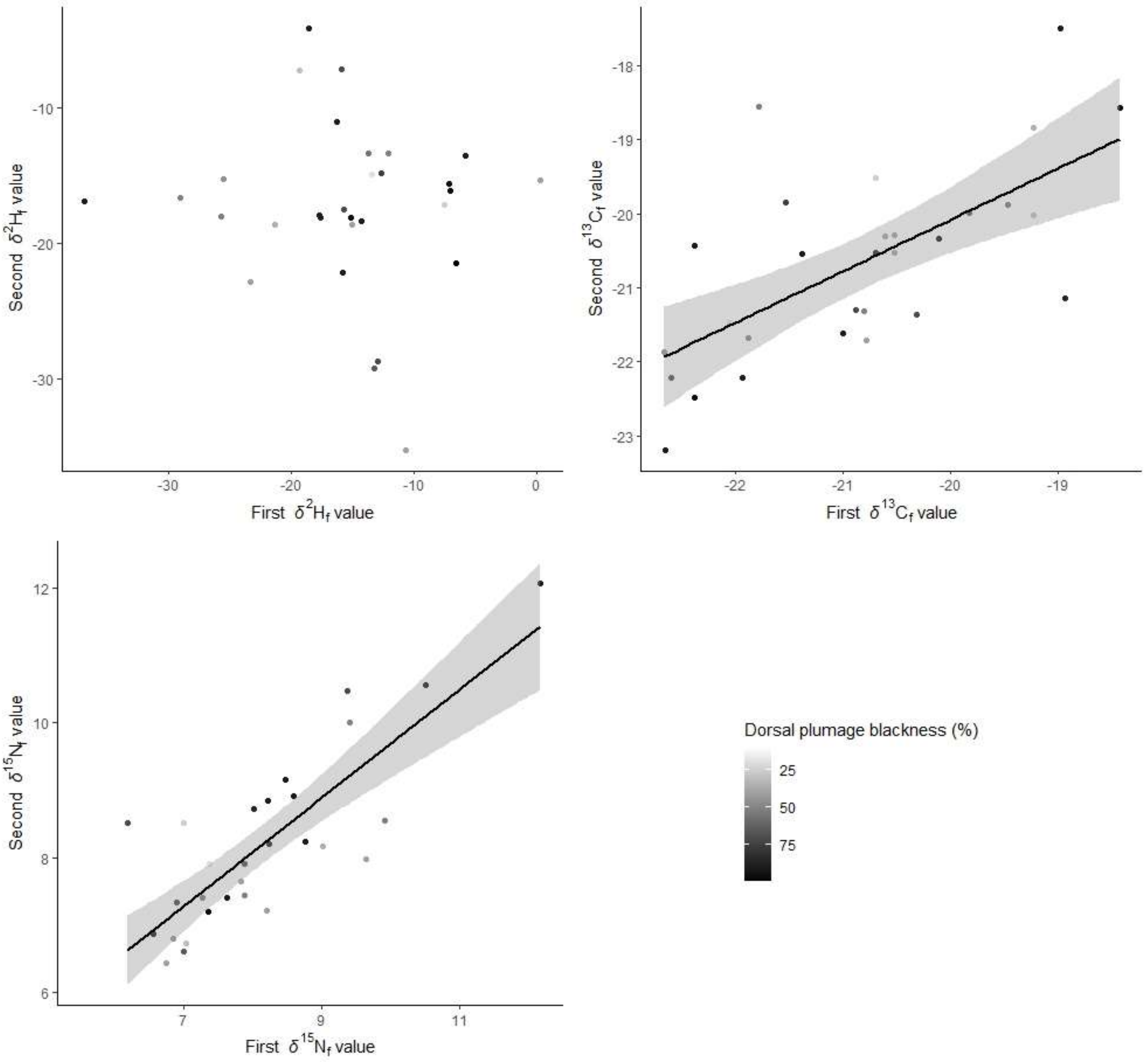

ESM2, Figure 3. Associations between successive isotope measurements within individuals (in ‰). Significant associations indicated with black regression lines. Data points are coloured according to individual plumage blackness in % as recorded during the first capture; lighter colour indicates blacker individual and darker colour browner individual.

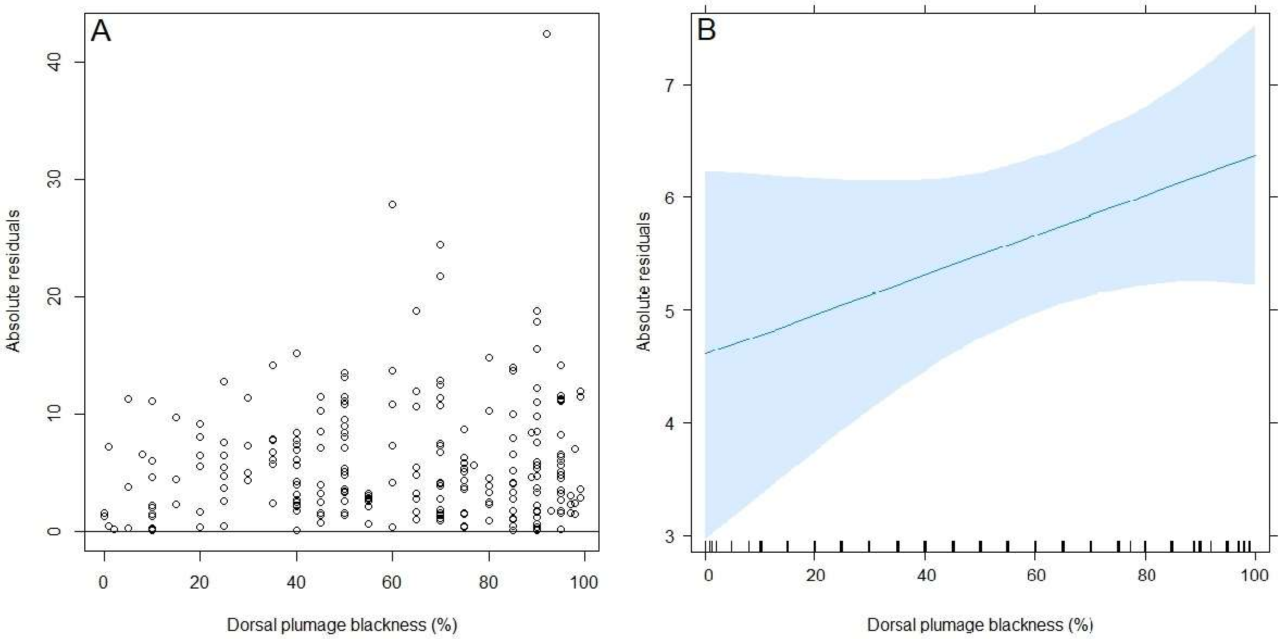

ESM2, Figure 4. In order to test if variation in δ^2H_f values increases with increasing plumage blackness, the absolute values of residuals of model δ2Hf ~ Plumage blackness were regressed against the values of plumage blackness. There was a weak but non-significant positive tendency for increasing variation in δ^2H_f values with increasing blackness (Slope estimate: 0.018 ± 0.01, t = 1.4, p = 0.15). **A.** Absolute residuals plotted against plumage blackness values. **B.** Effect plot giving predicted values of absolute residuals for given values of dorsal plumage blackness.

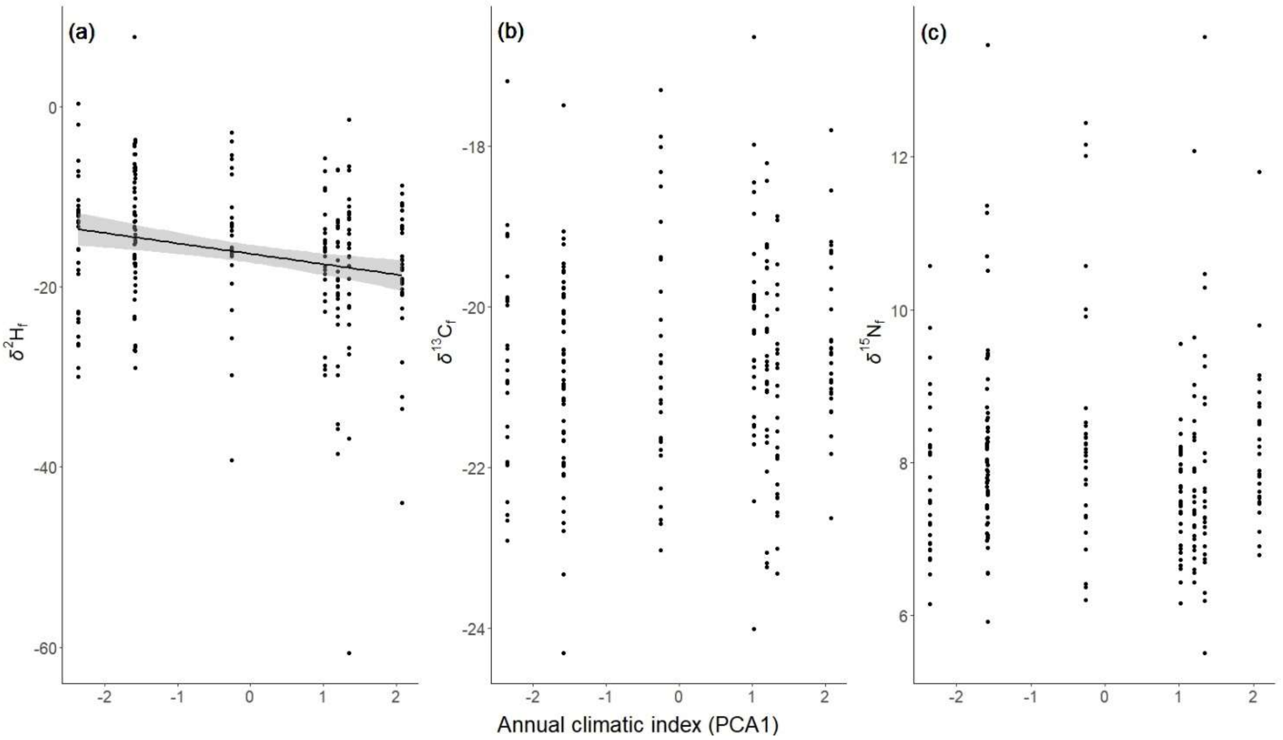

ESM2, Figure 5. Associations between PC1 of the annual climatic indices and the three different isotope (a=Hydrogen, b=Carbon, c=Nitrogen IN ‰) values in the feathers of individual pied flycatcher males. Significant association is indicated with black regression line.
